# Modern Approach to Nodal T-Cell Lymphomas

**DOI:** 10.1097/PAP.0000000000000492

**Published:** 2025-04-10

**Authors:** Sarah L. Ondrejka, Laurence de Leval

**Affiliations:** * Pathology and Laboratory Medicine Institute, Cleveland Clinic, Cleveland, OH; † Department of Laboratory Medicine and Pathology, Institute of Pathology, Lausanne University Hospital and Lausanne University, Lausanne, Switzerland

**Keywords:** international consensus classification, diagnosis, lymph node, peripheral T-cell lymphoma; not otherwise specified (NOS), follicular helper T-cell lymphoma, anaplastic large cell lymphoma, flow cytometry, high-throughput sequencing, reactive mimics, immunohistochemistry

## Abstract

In recent decades, there have been many meaningful contributions to the pathology literature with respect to T-cell lymphoma pathogenesis and biology and improved diagnostics. We know more about disease classification, clinical characteristics, immunophenotype, and genetics than ever before, and yet diagnosis of nodal T-cell lymphomas continues to be a challenging exercise. Complicating interpretation are the many non-neoplastic mimickers of peripheral T-cell lymphoma including drug effects, viruses, autoimmune, and idiopathic conditions, that must be considered when faced with an abnormal lymph node biopsy. The number of immunohistochemical stains required to make a diagnosis of T-cell lymphoma is not standardized and may be exhaustive, requiring judicious use of tissue sections. Clonality studies may contribute to the diagnosis, though questions remain about test modality, when to exercise interpretive caution, and what to do if a clone cannot be demonstrated. Use of next generation sequencing in the diagnosis of nodal T-cell lymphomas is increasing, but how the data can be practically applied to diagnosis is still under examination. The goal of this paper is to consider nodal T-cell lymphoma diagnosis and classification in a modern context, using a question-and-answer format to capture the interest of the reader and address common pathology consultation queries.

The recently published International Consensus Classification^1,2^ provides a framework for lymphoma diagnosis and highlights some relevant changes for peripheral T-cell lymphomas (PTCLs) including the unification of follicular helper T-cell lymphoma (TFH lymphoma) as a single entity that encompasses 3 subtypes: angioimmunoblastic-type (angioimmunoblastic T-cell lymphoma), follicular-type, and not otherwise specified (NOS). Primary nodal Epstein-Barr virus+ T-cell/NK-cell lymphoma, which was introduced in the 2017 WHO classification as a variant of peripheral T-cell lymphoma, NOS, is now considered a provisional entity. Within anaplastic large cell lymphoma, ALK-negative (ALK-negative ALCL), *DUSP22*-rearranged (*DUSP22*-R) ALK-negative ALCL is defined as a genetic subtype of systemic ALK-negative ALCL. Other published updates in T-cell lymphoma diagnosis apply to extranodal entities of T-cell lymphoma including those arising primarily in the gastrointestinal tract or skin and soft tissue, and are beyond the scope of this review.^1^ Despite subtle differences in terminology the 5th edition of the WHO classification (WHO-HAEM5) applies a similar rubric, converging on a fundamental understanding of TFH lymphoma and acknowledging the molecular heterogeneity within ALK-negative ALCL.^3–5^

Once the diagnosis of a nodal T-cell lymphoma is established, the boundary between some entities is sometimes blurred. For example, differentiating anaplastic large cell lymphoma, ALK negative, or adult T-cell leukemia/lymphoma from peripheral T-cell lymphoma, not otherwise specified, requires many considerations given the morphologic and immunophenotypic overlap. Establishing a follicular helper T-cell immunophenotype may help in diagnosis of a follicular helper T-cell (TFH) lymphoma, but appropriate diagnosis of this entity requires more than expression of TFH antigens. Each disease entity may encompass a broad spectrum of histopathologic features and slight variations to the immunophenotype, so a wide recognition of the subtypes of peripheral T-cell lymphoma is necessary. Finally, small-volume biopsies are increasing in popularity but create an obstacle to proper diagnosis and classification due to insufficient tissue for histologic review and ancillary studies, and the associated risk of diagnostic error. It is important to bear in mind that lymph nodes may be secondarily involved by mature T-cell neoplasms that are more typically found in the peripheral blood or bone marrow, such as T-cell prolymphocytic leukemia or adult T-cell leukemia/lymphoma (ATLL), or by disseminated T-cell lymphomas that are primary to extranodal locations such as skin (mycosis fungoides) or the intestine. These topics will be periodically discussed as they pertain to the differential diagnosis of nodal T-cell lymphoma.

The focus of this paper is to provide a practical approach to lymph node-based PTCL and to concentrate on the entities that are considered primary to lymph nodes, mainly, ALCL, TFH lymphoma, PTCL, not otherwise specified (NOS), and primary nodal EBV-positive T-cell/NK-cell lymphoma (Table 1). We begin with when to consider a T-cell lymphoma in a lymph node biopsy rather than a reactive proliferation, discuss immunophenotypic, flow cytometric, and molecular adjuncts to diagnosis, and consider difficulties in the differential diagnosis among T-cell lymphoma entities.

**TABLE 1:** Summary of Disease Characteristics of the Primary Nodal T-Cell Lymphoma Entities

Entity	Epidemiology	Morphology	Immunophenotype	Genetics
ALK+ ALCL	16% of PTCL M>F Median age 30-35 y	Hallmark cells Variable patterns: Common Lymphohistiocytic Small cell Hodgkin-like Spindle cell	CD30+, ALK+, EMA+, often CD2+, CD4+, CD43+; usually loss of several T-cell antigens (CD3, CD5, CD7), EBV-, cytotoxic markers+phospho-STAT3+	*NPM1*::*ALK1* fusion is most common, *ALK1* has many alternative partners JAK-STAT signaling activation
ALK- ALCL	8%-9% of PTCL M>F Median age 54 y	Hallmark cells Common pattern	CD30+ (uniform, strong), loss of some T-cell antigens, EMA +/-, EBV-, cytotoxic markers +/-, phospho-STAT3 + or -	*DUSP22*-R (20-30%) *TP63*-R (2-8%) JAK-STAT signaling activation± mutations Rarely other tyrosine kinase domain gene fusions
TFH lymphoma AI-type Follicular-type NOS	27% of PTCL M>F Median age 60-65 y	AITL-type: patterns I, II, III, often diffuse with polymorphic inflammatory infiltrate and FDC expansion Follicular-type: Follicular lymphoma-like or PTGC-like NOS: Diffuse and no FDC expansion or T-zone pattern	CD3+, CD4+, TFH positive (PD1, CXCL13, ICOS, CD10, BCL6), loss of other T-cell markers (CD7 or CD5), scattered EBV positivity CD21 helps identify FDC meshwork pattern Reactive CD8+ cells can be relatively abundant	Mutations in epigenetic regulators (*TET2*, *DNMT3A*) Hotspot *RHOA* G17V mutation and *IDH2* R172 mutations Fusions involving *CD28* and *ICOS* or *CTLA4, ITK*::*SYK* Gain-of-function mutations (*PLCG1*, *CD28*, *CD28*, *FYN*, *PIK3*, *CARD11*)
PTCL, NOS	26%-27% of PTCL M=F Median age 60 y	Variable cytology, small irregular or medium-large lymphocytes Diffuse growth pattern Variable microenvironment Lennert pattern <10%	Positive for pan-T-cell antigens (CD3, CD2, CD5, CD7) with reduced/absent expression of some antigens CD4+ CD8+, CD4+CD8+ or CD4-CD8-, CD30+/-, TCRαβ>γδ, LEF1+, cytotoxic +/-	*CDKN2A* and *PTEN* deletions, *TP53* alterations Low prevalence of *IRF4*, *TP63*, *VAV1*, *FYN*, *CD28* fusions Molecular subgroups defined by gene expression profiling: PTCL-TBX21 PTCL-GATA3
Primary nodal EBV+ T/NK-cell lymphoma	Rare, 1%-10% of PTCL with geographic variation and a higher incidence in Asian countries Elderly patients	Monomorphic large cell morphology Lack of angiocentricity or necrosis	CD3+, CD5+/-, activated cytotoxic phenotype, EBV diffusely+with type II latency pattern	Recurrent mutations in *TET2*, *DNMT3A*, *STAT3*, *PIK3CD*, *DDX3X* 14q11.2 loss

AI indicates angioimmunoblastic; ALCL, anaplastic large cell lymphoma; ALK, anaplastic lymphoma kinase; EBV, Epstein-Barr virus; NOS, not otherwise specified; PTCL, peripheral T-cell lymphoma; PTGC, progressive transformation of germinal centers; R, rearranged.

## AT WHAT POINT DO WE BELIEVE THAT A BIOPSY IS T-CELL LYMPHOMA RATHER THAN A REACTIVE PROLIFERATION?

**FIGURE 1:**
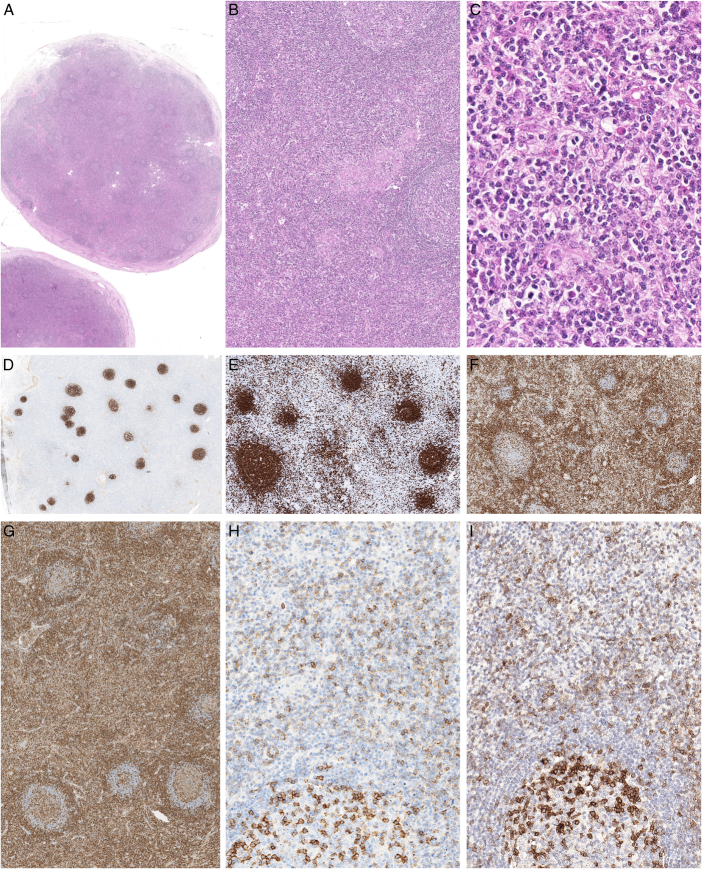
Reactive lymph node with paracortical expansion in a 74-year-old man with chronic thoracic and cervical lymphadenopathy. A, A 2 cm cervical lymph node was excised and showed overall preserved architecture with capsular fibrosis. B, Hyperplastic follicles were separated by enlarged paracortical areas containing histiocytic aggregates. C, The paracortical areas contained a mixed lymphoid population with scattered eosinophils. D, CD21 showed tight follicular dendritic cell meshworks restricted to the follicles. E, CD20 stained the follicles and a minority of the cells outside. CD3 stained the paracortical areas (F) where the lymphoid cells were predominantly CD4+ (G). Many cells in the paracortex were positive for PD1 (H) or ICOS (I), with a staining intensity lower than in the follicular helper T cells in the germinal centers. This biopsy was initially mistakenly interpreted as follicular helper T-cell lymphoma, but clonality and sequencing analyses failed to demonstrate a monoclonal TR gene rearrangement or somatic mutations.

The most important consideration when making a diagnosis of a nodal T-cell lymphoma is to be certain that a reactive lymphoproliferation is not mistaken as a neoplasm. It is recommended that the presence of several features associated with malignancy including the clinical presentation, lymph node histology, immunophenotype, and genetics are present and in alignment with the diagnosis (Table 2). Paracortical hyperplasia is one of the most common and nonspecific reactive patterns of lymphadenopathy and is composed of interfollicular expansion by histiocytes, plasma cells, eosinophils, immunoblasts, and vascular proliferation. As T-cell lymphomas can exhibit polymorphic inflammation with a T-zone or interfollicular pattern and eosinophilia, paracortical hyperplasia including with atypical features (partial effacement, occasionally a small clonal T-cell gene rearrangement) can be mistaken for lymphoma (Fig. 1). Paracortical hyperplasia can be seen in the setting of recent viral infection, vaccination, anticonvulsant therapy or other drug-induced lymphadenopathies (phenytoin, carbamazepine, amlodipine, fluoxetine, allopurinol, sulfonamides, methimazole, lamotrigine, gabapentin, nevirapine, monoclonal antibody therapy, among others).^6^ Reactive lymphoid proliferations usually lack complete architectural effacement of the lymph node and the polymorphous infiltrate is composed of recognizable immune cell types without significant atypia. The lymph node sinuses should remain patent and B-cell follicles are not widely separated and are composed of primary and secondary follicles in different stages of evolution. In a reactive lymph node, follicular dendritic cell meshworks are intact without attenuation or expansion. In contrast, complete effacement of the architecture with compressed or obliterated lymph node sinuses and the presence of a cytologically abnormal lymphoid population is more suggestive of lymphoma.^7^ Diffuse paracortical hyperplasia with subtotal effacement of the lymph node architecture and immunoblastic proliferation is the most common morphologic pattern in viral lymphadenitis. Infectious mononucleosis represents a common mimicker of T-cell lymphoma; it contains a robust interfollicular infiltrate of immunoblasts and isolated Reed-Sternberg-like cells and occasionally areas of necrosis, in a background of a high proportion cytotoxic CD8+ T cells.^7,8^ Caution is advised in that a small clonal T-cell receptor gene rearrangement may be detected in the setting of benign, self-limited infectious mononucleosis.^9,10^

**TABLE 2:** Features of Malignancy in Nodal T-Cell Lymphoma

Feature	Favors malignant	Favors benign
Clinical features	B symptoms, systemic lymphadenopathy, syndromic features of AITL (polyclonal hypergammaglobulinemia, hemolytic anemia, skin rash, rheumatologic symptoms) Hemophagocytic lymphohistiocytosis (although this has many underlying etiologies) Elevated LDH	Localized lymphadenopathy Lack of B symptoms Medication record: anticonvulsants, amlodipine, fluoxetine, monoclonal antibody therapy Recent vaccination
Lymph node histology/architecture	Effaced Proliferated and disrupted follicular dendritic cell meshworks Open subcapsular sinus with perinodal infiltrate Sinusoidal involvement by large neoplastic cells Hallmark cells, atypical lymphoid cells (ie, irregular nuclei, clear cells)	Paracortical expansion Preserved follicle density Open sinuses Polymorphous interfollicular infiltrate (small lymphocytes, histiocytes, plasma cells, eosinophils) Immunoblasts near high endothelial venules
Flow cytometry	Loss of surface CD3 Loss of CD7 or CD5 CD3+/CD4+/CD10+ T cells Monotypic TRBC1/TRBC2	Elevated CD4:CD8 ratio without loss of other T-cell antigens Identification of a very small TFH subset
Immunophenotype	Strong/diffuse CD30 positivity Relative increase in PD1+/CD10+/ICOS+/CXCL13+/BCL6+ T cells Abnormal CD21+ FDC meshwork pattern Loss of T-cell markers Cross-antigen expression (eg. CD20+ T cells, CD4+/TIA+ T cells)	CD30+ immunoblastic proliferation Relative increase in T cells expressing less than 2 TFH markers or positive for TFH markers with a dim level of expression Preserved CD21+ FDC meshwork pattern Retention of T-cell markers
Genetics	Clonal TR rearrangement (beta or gamma or both) Monoclonal IGH or IGK rearrangements may be detected in T-cell lymphoma with a B-cell component (TFH lymphomas) Hotspot pathogenic mutations (ie, *RHOA G17V*, *IDH2*, etc.) or pathogenic mutations in known drivers of malignancies Rearrangements of *DUSP22*, *TP63*, *ITK*::*SYK*, *CD28*::*ICOS*, *CD28*::*CTLA4*	Polyclonal TR rearrangement or isolated tube C in TR-beta testing Caution is needed if only *TET2* or *DNMT3a* variants are identified, as these may indicate nonspecific clonal hematopoiesis

Caution is advised if a specimen has only a few characteristics favoring malignancy.

Drug-induced lymphadenopathy has been described especially in the setting of anticonvulsant therapy and causes generally less prominent architectural distortion as it also encompasses germinal center hyperplasia, eosinophilia, and immunoblastic proliferation. Depending on the duration of drug therapy from hypersensitivity reactions to longer duration effects, the lymph node may have features from focal obliteration with eosinophilia and necrosis, to a burned-out phase with follicular depletion.^11^ Knowledge of the medical chart is helpful since certain immune-mediated drug reactions such as drug rash with eosinophilia and systemic symptoms (DRESS) is characteristically accompanied by fever, rash, elevated liver enzymes, and C-reactive protein, occurring 1 to 8 weeks after drug introduction.^12^

**FIGURE 2:**
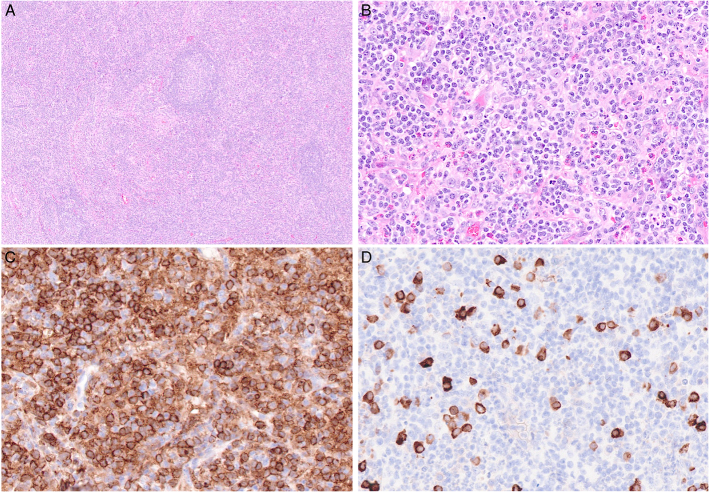
IgG4 lymphadenopathy. This lymph node is characterized by marked enlargement of the paracortex (A) which shows prominent vessels and a polymorphous infiltrate including large lymphoid cells, eosinophils, and plasma cells (B). Many lymphoid cells are CD4+ (C). Many plasma cells are IgG4+ (D).

IgG4-related lymphadenopathy occurs in the spectrum of IgG4-related disease, affecting adult patients with mass-forming lesions or organ symptoms of fibrosclerosis and elevated serum IgG4. Involvement of lymph nodes can mimic a TFH lymphoma particularly in cases characterized by diffuse interfollicular expansion with plasma cells, immunoblasts and plasmablasts, and eosinophils, and occasionally follicular regression (Fig. 2).^13^ This can appear similar to a histologic pattern II angioimmunoblastic T-cell lymphoma, with regressive germinal centers and inflammatory interfollicular proliferation of immune cells.^14^

Mimics of PTCL include inborn errors of immunity with lymph node manifestation such as autoimmune lymphoproliferative syndrome (ALPS), due to mutations in the *FAS/FAS-L* and defective apoptosis. These patients may demonstrate lymph node enlargement with paracortical expansion by mature, double negative (CD4-/CD8-) cytotoxic αβ T cells and increased interfollicular vascularity. Distinguishing features of ALPS lymph nodes from PTCL include a spectrum of reactive germinal center changes, an increase in CD5-coexpressing B cells, and florid plasmacytosis and considering the clinical context. Significant increases in double negative (CD4-/CD8-) T cells with an accompanying polyclonal B-cell lymphocytosis by peripheral blood flow cytometry may be identified in patients with ALPS.^15^

**FIGURE 3:**
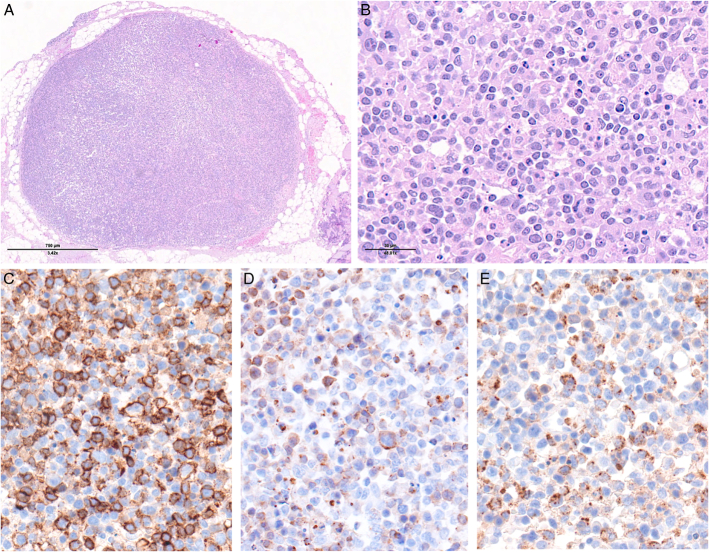
Kikuchi-Fujimoto. A, The lymph node shows a completely effaced architecture. B, It contains a lymphohistiocytic infiltrate with large atypical lymphoid cells. CD8 stains many of the lymphoid cells including the large ones (C) and perforin demonstrates a cytotoxic phenotype (D). E, Many histiocytes express myeloperoxidase.

Histiocytic necrotizing lymphadenitis (Kikuchi-Fujimoto lymphadenitis) causes sometimes near-total lymph node effacement by paracortical hyperplasia of histiocytes and activated T cells that can be large and immunoblast-like with a high Ki-67 proliferative fraction, with confluent areas of necrosis and karyorrhexis, overall worrisome for T-cell lymphoma (Fig. 3). An important clue is the clinical context of Kikuchi-Fujimoto disease often occurring in young women with cervical lymphadenopathy. Recognition of features of Kikuchi-Fujimoto lymphadenitis such as crescentic, myeloperoxidase-positive histiocytes, increased plasmacytoid dendritic cells, and few/absent plasma cells and absent neutrophils can be helpful, as well as understanding the progression of histologic stages that occur. These include an early proliferative lesion with abundant atypical mononuclear cells, a necrotizing phagocytic lesion, and a late xanthomatous form.^16^ Since PTCL can be composed of large neoplastic lymphoid cells in some cases with intermixed histiocytes and areas of necrosis, it is important to keep Kikuchi-Fujimoto lymphadenitis in mind especially in smaller biopsies in which the full morphologic and geographic spectrum of lymphadenitis may not be revealed.

**FIGURE 4:**
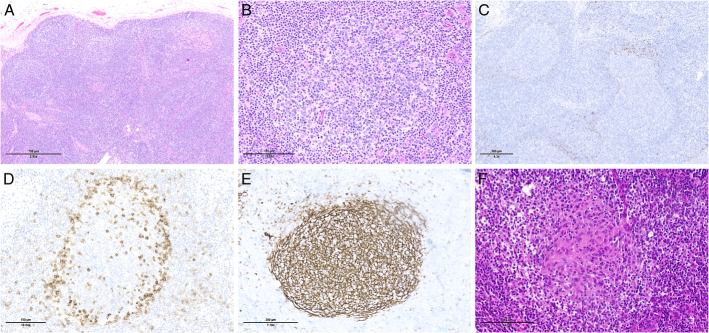
Follicular helper T-cell lymphoma, angioimmunoblastic-type, patterns 1 (A–E) and 2 (F). In pattern 1, the low-power view of the lymph node shows large reactive germinal centers (A) with attenuated mantle zones and atypical clear cells at the periphery (B); IgD confirms paucity of residual mantle cells (C), ICOS stains the atypical cells forming a rim surrounding germinal center (D) and CD21 shows a tight follicular dendritic cell meshwork restricted to the germinal center (E). In pattern 2, atypical clear cells are seen in association with a regressed follicle (F).

Conversely, there are some types and growth patterns of nodal T-cell lymphoma that may not be so obviously recognized and could be considered false-negatives. TFH lymphoma, AI-type pattern 1 features large hyperplastic germinal centers, often with absent or attenuated mantle zones and perifollicular abnormal clear cells merging into a polymorphous infiltrate with increased vascularity in the paracortex, and pattern 2 is characterized by regressed follicles mimicking Castleman disease (Fig. 4). Follicular dendritic cell (FDC) meshworks are not expanded and are relatively intact, unlike the FDC pattern seen in other types of TFHL AITL-type.^17^ Examples of TFH lymphoma AI-type have been described including several presented at the 2022 EAH4P workshop with low tumor burden and indolent, paucisymptomatic clinical behavior despite disseminated low-level disease. The lymph nodes from these patients demonstrated mostly preserved architecture with expanded mantle zones and follicular involution, and subtle infiltrates of abnormal TFH cells around the outer edge of follicles or in the paracortex.^18^ Regarding PTCL, NOS, some cytologic variants are composed of predominantly small cells. Clues to diagnosis include an effaced growth pattern, abnormal phenotype, and molecular data.

## HOW DOES FLOW CYTOMETRY ASSIST IN A DIAGNOSIS OF T-CELL LYMPHOMA?

Flow cytometric immunophenotyping is a sensitive diagnostic tool that contributes to a diagnosis of T-cell lymphoma by analysis of a fresh single-cell suspension of lymph node for deviations in antigen expression from normal T cells and, in some cases, demonstration of clonality. Careful attention to tissue processing is necessary to ensure cellular disaggregation while minimizing cell loss and nonspecific antibody binding, and optimizing antigenicity and viability.^19^ The T-cell immune response is complex and requires understanding of the major physiological T-cell subsets in addition to subtle alterations in antigen expression that can expand in various disease states. For example, prior work focused on analyzing T-cell subsets in spleen demonstrated small reproducible subsets of normal CD4+CD7- T cells (late memory T cells), subsets of T cells with altered CD5 expression or intensity (naive CD8+ cells), and minor CD4+ CD8+ populations.^20^ Successful performance of T-cell analysis by flow cytometry requires skilled technical staff, sensitive well-maintained instrumentation, tight processing protocols, and careful choice of antibodies and tagged fluorochromes in panel design.^21^ Lymphoma cells may exhibit increased autofluorescence as compared with background/normal T cells, and thresholds for positivity or negativity must be carefully considered. Nonetheless, features suggestive of T-cell neoplasia including a significantly abnormal CD4-to-CD8 ratio beyond what is considered physiological, a lack of expected T-cell markers (eg, CD2, CD5, CD7), CD10 expression, or abnormal staining intensity for any T-cell marker can be adjunctively helpful to making a diagnosis. In expert hands, sensitivity reaching as high as 92% for immunophenotypic aberrancy in PTCL samples can be achieved by cluster analysis and comparison of populations to laboratory-established reference ranges for known non-neoplastic T-cell subsets.^22^ In a large retrospective analysis of solid tissues comparing flow cytometry with histopathologic diagnosis that included 73 cases of nodal T-cell lymphoma, there was agreement between flow cytometry interpretation and final histopathology in 66.7% to 88.2% of cases depending on the T-cell lymphoma entity, and results were comparable even in cases with <20% neoplastic cells. Cases with nonagreement were attributed to the inability to detect an aberrant population by flow cytometry, suggesting that an abnormal population is helpful when present, but a normal flow cytometry result does not exclude the diagnosis of T-cell lymphoma.^23^

**FIGURE 5:**
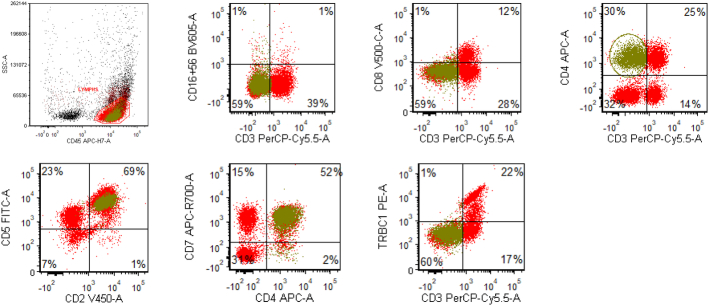
Flow cytometry dot plots of an example case of follicular helper T-cell lymphoma. Compared with normal T cells (red), the abnormal T cells (green) lack surface CD3, and are positive for CD4, CD2, CD5, and CD7. Although TRBC1 appears uniformly negative, it cannot be interpreted in the setting of surface CD3 loss.

Prior studies of AITL by flow cytometry have provided predictable aberrant patterns to look for in assessment of TFH lymphoma. The neoplastic T cells vary in proportion to the background non-neoplastic T cells, ranging from 23% to 29% of lymphocytes. Absent or dim positivity for surface CD3 is a significant immunophenotypic aberrancy for mature T cells, which in combination with antigens such as CD4 and/or CD5 would still permit recognition as T cells rather than NK cells (which would also lack surface CD3 and express CD2 and CD7).^21^ The majority of cases express CD2, CD4,CD5, CD10+/-, and CD45, and negative or dim surface CD3. Absence of CD7 expression on a significantly expanded subset of CD4+ T cells is another common abnormal feature.^24,25^ Identification of CD3-/dim CD4+ aberrant T cells by flow cytometry can be a helpful feature in excluding morphologic mimics of TFH lymphoma such as reactive hyperplasia or Hodgkin lymphoma (Fig. 5).^26,27^ It is important to mention that CD10 is present on a small subset of normal TFH cells which make up a minor component of reactive germinal centers, so very small populations of CD4+CD10+ T cells without other phenotypic aberrancies should be interpreted with caution and with histologic correlation. Flow cytometry is not always able to detect an immunophenotypically abnormal T-cell population in AITL/TFH lymphoma; in one large study of 155 cases, an aberrant T-cell population was detected in 97 cases representing 0.5% to 90% of lymphocytes.^24^ Another caveat is that rarely cases of TFH lymphoma, AI-type or PTCL have occurred in association with EBV+ diffuse large B-cell proliferations interpreted as DLBCL, or with a monoclonal B-cell expansion that can take a number of forms and may be detected by flow cytometry. In such cases, the abnormal T-cell subset may be overlooked, especially if comprising a low percentage of overall cellularity, or if it cannot be detected at all and a monoclonal B-cell population is detected, the working diagnosis may stray farther from a PTCL.^28,29^

Analysis of the T-cell receptor beta chain constant region (TRBC) by flow cytometry can be a useful method for evaluation of T-cell clonality, since its 2 mutually exclusive isoforms (TRBC1 and TRBC2) can be interpreted in a manner analogous to kappa and lambda light chains. Several studies have demonstrated the utility of adding TRBC1 and/or TRBC2 to an 8-color or 10-color flow cytometry panel with other T-cell antigens to evaluate clonality in αβ T cells.^30–32^ A limitation to this approach is that γδ T cells lack TRBC1 and TRBC2, so gating analysis is limited to surface CD3 expressing, αβ T cells in order to avoid overinterpretation as a TRBC1-negative clonal T-cell population.^33^ In some specimens, single staining for TRBC1 can produce monotypic TRBC1-dim subsets that can be difficult to interpret within laboratory-defined thresholds (eg, <15% or >85% TRBC1-positive events).^33^ A small subset of dim monotypic TRBC1 expressing T cells can be incidentally detected in patients with reactive or comorbid medical conditions and should be interpreted with caution.^34^ Larger T-cell populations with dim monotypic TRBC1 have been shown by additional studies to represent TRBC2-positive T cells.^31^ The addition of TRBC2 to the panel can help to resolve an abnormal subset in a substantial proportion of cases. Some mature αβT-cell neoplasms that might appear TRBC1-negative on single staining (and therefore possibly TRBC2 restricted), may prove to be negative for both TRBC1 and TRBC2, suggesting that dual staining may provide more reliable information for determination of TRBC isoform expression.^31^

## WHAT IS THE BEST STEPWISE APPROACH FOR DIAGNOSING NODAL T-CELL LYMPHOMA?

**FIGURE 6:**
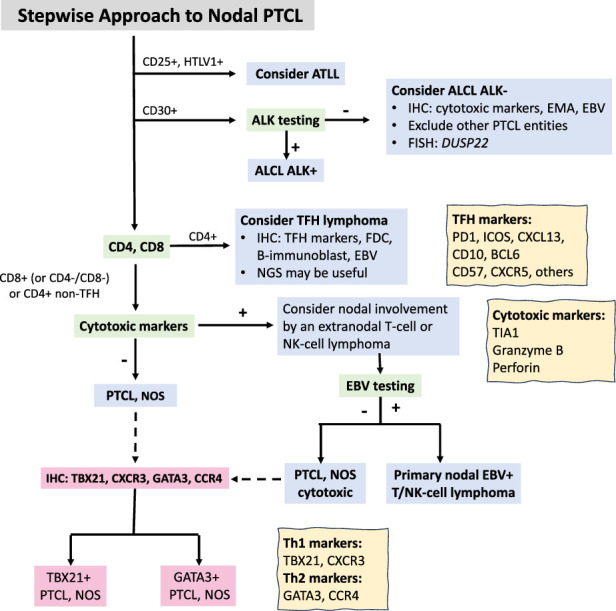
Algorithm for the workup and classification of nodal PTCLs. Once a T-cell lymphoma is established, this hierarchical orientation to subclassification of specific entities is helpful to the pathologist. Modified with permission from Campo et al.^1^ Adaptations are themselves works protected by copyright. So in order to publish this adaptation, authorization must be obtained both from the owner of the copyright in the original work and from the owner of copyright in the translation or adaptation.

**FIGURE 7:**
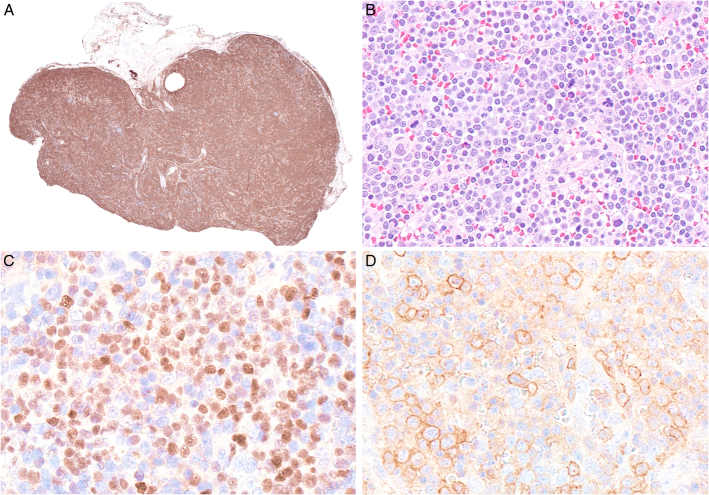
Adult T-cell leukemia/lymphoma, lymphomatous form. A, The lymph node is diffusely involved by a CD3+ population. B, The infiltrate consists of large pleomorphic atypical cells with numerous mitoses. The cells are positive for FOXP3 (C) and CD25 (D).

To accommodate an initial differential diagnosis that is comprehensive of T-cell lymphoma, we refer the reader to an algorithm for the workup and classification of nodal PTCLs (Fig. 6, adapted from Campo et al^1^) which provides a general framework for routine use. It begins with consideration of HTLV1 status which would contribute strongly to classification as ATLL despite a wide diversity of morphologic and immunophenotypic features that nearly match any other subtype of PTCL without HTLV1 association (Fig. 7).^35^

The approach to immunohistochemistry may consist of an initial limited panel of stains to assist in excluding B-cell lymphomas, Hodgkin lymphoma, and reactive processes from suspected T-cell lymphoma. This might include a few T-cell stains initially (CD3, CD5, perhaps CD4, and CD8 upfront if suspicion is high), CD10, CD20, CD21, CD30, CD45, PAX5 followed by additional T-cell antigens (CD2, CD4, CD7, CD8, TFH markers, cytotoxic markers, TCRγ, TCRβF1), specialized stains (ALK1, TCL1, TdT, CD56), additional FDC meshwork stains, and EBER in situ hybridization.^36^ CD3 is quite robust by immunohistochemistry and serves as an initial starting point with additional markers of T-cell subsets including CD4, CD8, and pan-T-cell markers CD2, CD5, and CD7 comprising a backbone for initial characterization.

**FIGURE 8:**
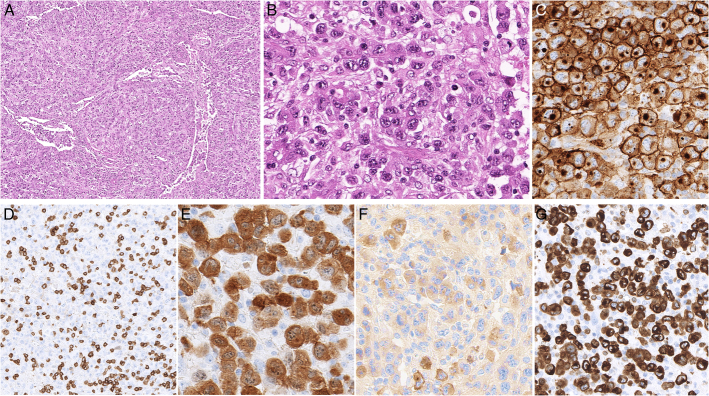
ALK-positive anaplastic large cell lymphoma. A, Low-power view of a lymph node showing diffuse involvement and intrasinusoidal dissemination. B, High-power view showing large anaplastic cells. C, The cells show strong homogeneous paranuclear dot-like and membranous CD30 expression. D, CD3 stains small reactive T cells and is negative in the large neoplastic cells. E, Nuclear and cytoplasmic positivity for ALK reflects a t(2:5) *NPM1::ALK* fusion. The neoplastic cells are positive for EMA (F) and perforin (G).

**FIGURE 9:**
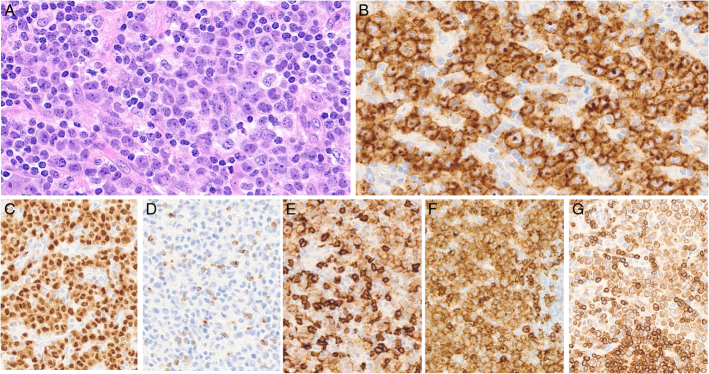
*DUSP22*-rearranged ALK-negative anaplastic large cell lymphoma. A, The large neoplastic cells form cohesive sheets and comprise many hallmark cells. The lymphoma cells are strongly positive for CD30 (B), positive for MUM1 (C), negative for granzyme B (D), positive for CD8 (dim) (E), positive for CD2 (F), and show downregulated BCL2 expression (G).

CD30 is a key marker for the diagnosis of T-cell lymphomas and can be used as an initial branch point for further subclassification.^37,38^ If the pattern of CD30 staining is strong and uniform and suggestive of ALCL, ALK protein expression is the next step to establish a diagnosis of ALK+ versus ALK- ALCL. ALK+ ALCL may be positive for CD4, CD43, CD8 (smaller subset of cases), and cytotoxic molecules, and is commonly negative for CD3, CD5, and TCR proteins (Fig. 8).^38,39^ If ALK is negative, the differential diagnosis remains broad but includes ALK- ALCL, along with ATLL, mycosis fungoides with large cell transformation involving a lymph node, other extranodal T-cell lymphomas involving a lymph node, and a subset of PTCL NOS with CD30 expression in >80% of lymphoid cells. Cases of ALK- ALCL, should show strong and uniform expression of CD30 and resemble the common pattern of ALK-positive ALCL. Variant patterns of ALK- ALCL are not recognized.^2^ FISH testing for *DUSP22* rearrangement is recommended by the ICC and enables the identification of this subgroup, which is important biologically and for prognosis.^1,40^ ALK- ALCL with *DUSP22* rearrangement (Fig. 9) are usually negative for cytotoxic markers and EMA, and have unique cytologic features including less pleomorphism with uniform hallmark cells and doughnut cells.^40,41^ Strong and uniform LEF1 expression had a high positive and negative predictive value for *DUSP22* rearrangement in ALK-negative ALCL.^42^ The characteristic morphologic and immunophenotypic features that help identify possible *DUSP22*-rearranged cases further support this entity as a distinct clinicopathologic subset of ALCL. Like ALK+ ALCL, ALK-, ALCL typically has aberrant loss of pan–T-cell antigens (CD3, CD5, and CD7), while CD43 and CD2 are more likely to be positive.^42^ ALCL is characterized by sinusoidal growth initially until it may take over geographic regions of the lymph node (Fig. 8).^44^ Infiltrates of ALCL may be subtle and missed if high magnification is not used or misinterpreted as metastatic carcinoma if confirmatory immunohistochemistry is not applied.

**FIGURE 10:**
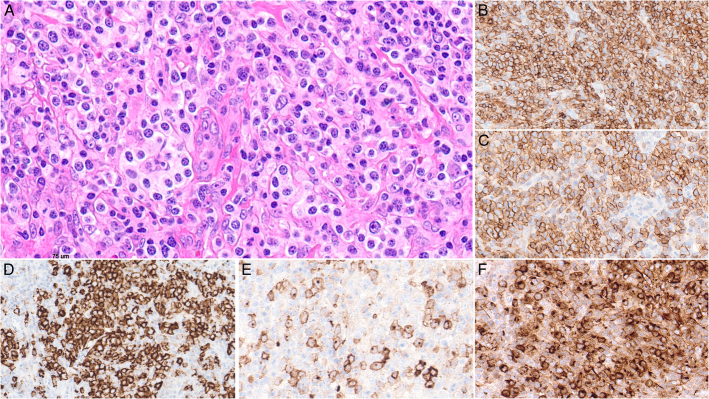
Follicular helper T-cell lymphoma. In this follicular helper T-cell lymphoma of the angioimmunoblastic type (A), the neoplastic cells are positive for CD4 (B), PD1 (C), ICOS (D), CD10 (E), and CXCL13 (F). The strong expression of follicular helper T-cell markers goes along with clear cell morphology of the neoplastic cells (A) in an *IDH2*-mutated case.

If ALCL is not an appropriate diagnosis, CD4 and CD8 immunohistochemistry provide another divergence to evaluate for a TFH lymphoma, since TFH lymphomas are derived from a functional subset of CD4+ T-helper cells with similarities in genetic landscape.^45–49^ The 5 markers recommended and most widely used to define this phenotype are PD1, CXCL13, ICOS, BCL6, and CD10,^50^ though other TFH markers include CXCR5, CD57, and SAP.^51^ A TFH immunophenotype is established when the lymphoma cells are positive for ideally 3 TFH markers, but a minimum of 2 TFH markers with convincing expression is acceptable in the right context (Fig. 10).^1^ In addition to assessing the immunophenotype, the distribution of TFH cells and intensity of staining should be considered, since TFH cells are normally present in lymph nodes in germinal centers and paracortex, and PD1 intensity may vary depending on the anatomic compartment. PD1 positive T cells may be increased in reactive conditions including viral lymphadenitis (Fig. 1).^52^ CD10 and CXCL13 may be positive in only a small subset of the neoplastic cells (Fig. 10E), and are generally less sensitive but more specific for a diagnosis of TFH lymphoma. PD1 and ICOS are much more sensitive, but less specific with overlap in reactive lymphadenopathies, and PD1 expression should be strong if it is used as a marker to rule in a TFH phenotype.^29,53^ Follicular dendritic cell meshwork stains are important to evaluate the lymph node architecture and lymphoma growth pattern; since AI-type and other types of TFH lymphoma have characteristic meshwork morphologies.^54^ It is important to recognize that TFH cells can be abundant in small B-cell neoplasms, specifically marginal zone lymphoma and follicular lymphoma, and represent a pitfall in the diagnosis of TFH lymphoma.^55,56^

**FIGURE 11:**
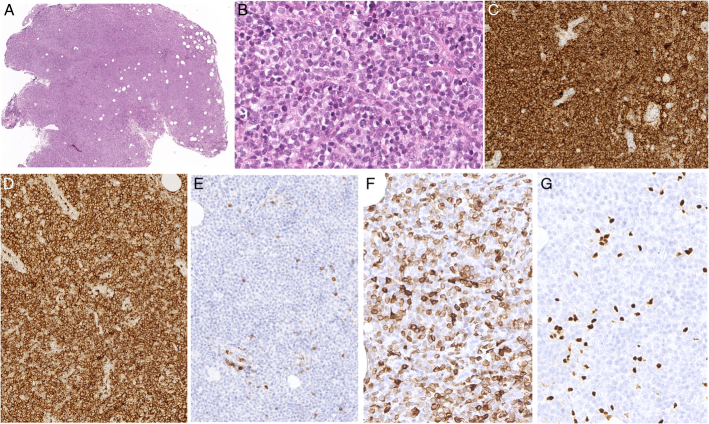
Peripheral T-cell lymphoma, NOS. The lymph node is diffusely involved by a proliferation of relatively monotonous medium-sized atypical lymphoid cells (A–B), which are positive for CD4 (C), CD2 (D), negative for CD7 (E), and aberrantly coexpress CD79a (F). G, Only few PAX5-positive cells are present. Stains for cytotoxic markers were negative (not shown).

If the T-cell lymphoma is CD8+, CD4-/CD8-, or CD4+ without a TFH immunophenotype, cytotoxic markers TIA1, granzyme B, and/or perforin should be performed with a positivity threshold of >50% tumor cells. In normal cells, the TIA1 protein is constitutively expressed in cytotoxic cells whereas perforin and granzymes are inducible upon activation,^57^ as such, cohorts of cytotoxic PTCL demonstrate TIA expression at a slightly higher rate.^58,59^ If cytotoxic molecules are present, nodal involvement by an extranodal PTCL or NK/T-cell lymphoma should be excluded. EBV testing can differentiate between a cytotoxic PTCL, NOS and primary nodal EBV+ T/NK-cell lymphoma. If cytotoxic molecules are negative, the diagnosis defaults to PTCL, NOS (Fig. 11).^1^ If a diagnosis of PTCL, NOS is established, an immunohistochemical algorithm could be performed to approximate the cell-of-origin from Th1 or Th2 functional subsets.^60^ The algorithm was proposed after gene expression profiling studies of PTCL, NOS identified 2 molecular subgroups with distinct transcriptomes and different prognosis: PTCL-TBX21 (Th1) and PTCL-GATA3 (Th2). Compared with PTCL-GATA3, PTCL-TBX21 has a better prognosis, except for a subset of the PTCL-TBX21 subgroup with a cytotoxic profile. PTCL-TBX21 has less genomic complexity, a higher frequency of epigenetic modifying genes, and enrichment in interferon and NF-ĸB pathways, while PTCL-GATA3 generally has a poorer prognosis, greater genomic complexity, and upregulation of the MYC-induced and PI3K-induced pathways.^61,62^ The proposed immunohistochemical algorithm begins with the determination of TBX21 expression and is followed by assessment of CXCR3, GATA3, and CXCR4, with thresholds for positivity for TBX21 and CXCR3 at 20%, and for GATA3 and CCR4 at 50%.^60^ A follow-up study showed that the algorithm could correctly classify 87% of cases compared with the digital GEP classification, and identified differences in microenvironment, tumor cell size, and immunophenotype between PTCL-TBX21 and PTCL-GATA3.^63^

If the diagnosis of PTCL NOS is under consideration and the lymphoma cells appear to be monotonous, medium-sized, blast-like, or expressing dual positivity or negativity for CD4 and CD8, the possibility of a T-lymphoblastic lymphoma needs to be excluded. TdT, CD34, and CD1a stains can be helpful. The differential diagnosis with T-lymphoblastic lymphoma (T-LBL) in a lymph node includes indolent T-lymphoblastic proliferation, usually forming aggregates of monotonous lymphoid cells with an immature thymocyte phenotype that are polyclonal and must be differentiated from T-LBL as clinical behavior is effectively benign.^64^

## HOW DOES EPSTEIN-BARR VIRUS TESTING IMPACT THE DIAGNOSIS OF T-CELL LYMPHOMA?

Examination for EBER RNAs by in situ hybridization or EBV by immunohistochemistry in either the neoplastic cells or the surrounding microenvironment impacts the diagnosis of nodal T-cell lymphoma and is a necessary step in diagnosis.

**FIGURE 12:**
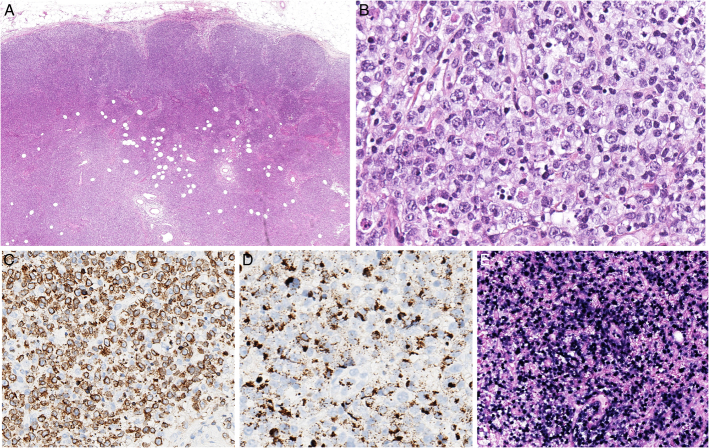
Primary nodal EBV+ T/NK-cell lymphoma. A, A lymph node biopsy in a 36-year-old man with chronic active EBV disease who developed lymphadenopathy shows an effaced architecture with infiltration of the hilar fat. The tissue infiltrate consists of large atypical lymphoid cells (B) which are positive for CD3 (C) and perforin (D). E, Most cells are positive for EBV by situ hybridization with EBER probes.

Primary EBV+ nodal T/ NK-cell lymphoma (Fig. 12) is an entity distinct from extranodal NK/T-cell lymphoma nasal type (ENNKTCL) that is listed as a provisional entity in the ICC classification. It is a primary nodal disease and must be distinguished from other T/NK EBV+ lymphoproliferative disorders that may infiltrate lymph nodes. Clinical criteria for diagnosis require exclusion of nasal involvement, while dissemination to extranodal sites such as bone marrow, liver, or other viscera is permitted. EBV testing is mandatory to demonstrate EBV positivity (usually latency pattern II) in virtually all the tumor cells. It can be distinguished from ENNKTCL by pathologic features including monomorphic large cell morphology, lack of angiocentric growth and necrosis, negativity for CD56 and positivity for CD8, and lineage corresponding to T cells more closely than NK cells. The tumor occurs in older adults from East Asia and has a dismal prognosis even when compared with ENNKTCL or PTCL, NOS.^65–68^ The differential diagnosis with ENKTCL comes up because cervical lymph node involvement by ENKTCL may be detected before identification of a nasal mass.^69^ Some cases of primary EBV-positive nodal T-cell lymphoma can have moderate to high expression of CD30, so EBER positivity in the majority of the tumor cells is a helpful diagnostic feature to exclude CD30+ PTCL, NOS, or ALK- ALCL.^38^

**FIGURE 13:**
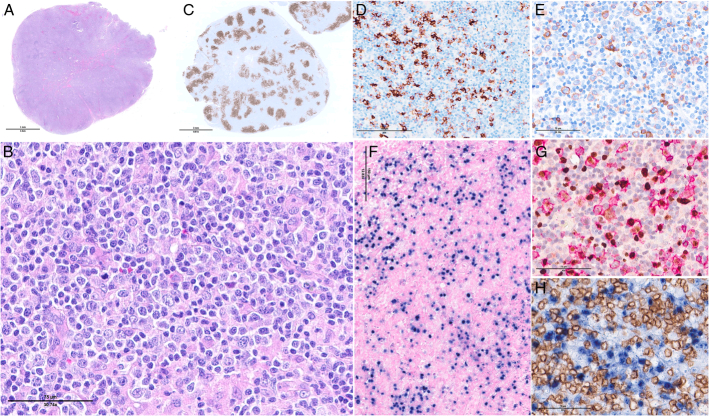
EBV-positive large B-cell proliferation in association with follicular helper T-cell lymphoma, angioimmunoblastic type. An enlarged inguinal lymph node in a 74-year-old woman with generalized lymphadenopathy has an effaced architecture (A) and contains a pleomorphic proliferation of medium sized and large lymphoid cells with abundant vessels (B). C, CD21 stains irregularly enlarged follicular dendritic cell meshworks. ICOS highlights atypical medium-sized cells (D) and CD79a stains many large blastic cells (E). F, Many cell are positive for EBV by in situ hybridization with EBER probes. Double stains for EBER and CD20 (brown and red, G) and EBER and CD3 (dark blue and brown, H) show that the EBV-positive cells belong to the B-cell lineage. Polyclonal IG gene rearrangements were found in this case.

A common feature of TFHL is the presence of EBV+ clonal B-cell and plasma cell proliferations including large B cells expressing CD30 and resembling Hodgkin/Reed-Sternberg (HRS) cells.^70,71^ The B-cell proliferation is polyclonal or clonal and heterogeneous, and TFHL especially AITL is described with a variable number of EBV+ B cells, often as isolated or small clusters of B blasts to focally confluent large B cells that can mask an underlying of TFH lymphoma (AITL) (Fig. 13).^72^ In some circumstances, TFH lymphoma of AI-type may have EBV-negative HRS-like cells or EBV-negative clonal plasma cell proliferations, so the absence of EBV does not argue against the phenomenon but is helpful when present.^71,73^ Some patients with TFH of AI-type can develop aggressive B-cell proliferations with latency patterns II or III, similar to those seen in immunosuppressed individuals.^72^ While previous publications have reported EBV-positive proliferations in PTCL, NOS, it is likely a lower occurrence than previously reported due to earlier definitions of PTCL, NOS that may have included cases of TFH lmphoma.^68^

## IN WHICH DIAGNOSTIC SITUATIONS IS NEXT GENERATION SEQUENCING MOST CONTRIBUTORY AND ARE CLONALITY STUDIES RECOMMENDED FOR ALL CASES?

T-cell receptor (TR) gene rearrangement studies have been useful as markers of lineage and clonality in T-cell neoplasms for many years. The BIOMED-2 primer PCR tubes were reported to detect clonal TR gene rearrangements in >90% of T-cell malignancies and had a reasonable rate of polyclonal testing in reactive lesions.^74^ Though this is not absolute, the complete IG/TR gene rearrangement pattern of a lymphoid malignancy might support lineage assignment. For example, TFH lymphoma AI-type might harbor both TR- and IG-rearrangements. Along those lines, detection of a solitary IG-rearrangement without a detectable T-cell clone does not completely exclude the possibility of a T-cell lymphoma. It should be noted that TR gene studies in reactive tissues may show clonal T-cell populations but these are usually small clones and are not synonymous with malignancy.^75,76^ Next generation sequencing-based technology for analysis of the TR gene has demonstrated high efficiency and reliability in detecting clonal rearrangements in T-cell lymphomas, but this is not (yet) in widespread use.^77,78^ Nonetheless, identification of a clonal TR gene rearrangement in the appropriate clinical and pathologic context can be very helpful in establishing a diagnosis of T-cell lymphoma, while a polyclonal pattern can support a reactive lymphoproliferation. An algorithmic study of T-cell lymphoma diagnosis in North America determined that after a tiered comprehensive immunohistochemistry approach, the addition of T-cell receptor PCR studies provided additional positive value to achieving a WHO 4th ed. diagnosis, yet gene rearrangement contributed to a change in only 8% of reviews.^36^

Targeted gene panels for high-throughput sequencing (HTS) have been adapted to formalin-fixed paraffin-embedded tissue, though classification of T-cell lymphoma is still primarily accomplished by morphologic assessment and immunophenotyping. Nonetheless, there are some diagnostic scenarios where clonality testing and HTS may be effective. For T-cell lymphoproliferations with HRS-like cells in a T-cell rich background, clonality analysis is useful because a monoclonal TR rearrangement supports a diagnosis of T-cell lymphoma and argues against CHL or B-cell lymphomas. Monoclonal IG-rearrangements may be variably demonstrated in CHL, nodular lymphocyte-predominant B-cell lymphoma, and T-cell/histiocyte rich large B-cell lymphoma as well as in TFHL with an associated B-cell component. Demonstration of mutations in genes commonly mutated in T-cell lymphomas (*CARD11*, *CD28*, *DNMT3A*, *IDH2*, *PLCG1*, *RHOA*, *STAT3*, and *TET2*) supports that diagnosis.^79^ Up to 80% of patients with TFHL have underlying clonal hematopoiesis, with shared *TET2* and/or *DNMT3A* mutations in the early progenitor cells as well as the lymphoma, so identification of these types of mutations without more specific variants (eg, *RHOA* and *IDH2*) may not be as meaningful to a diagnosis.^80–82^ Another scenario in which HTS can be helpful is in the evaluation of T-cell expansions with a TFH immunophenotype. In this setting, demonstration of a monoclonal TR gene rearrangement or somatic mutation in relevant genes is useful in the distinction between reactive versus neoplastic expansions of TFH cells. During evaluation of a TFH expansion, the identification of mutations in genes related to B-cell lymphomas favoring marginal zone or follicular lymphoma would potentially be a cause for re-examination of the specimen and help in supporting a reactive TFH proliferation.^79^ RNA-based expression profiling has emerged as a new tool for determining PTCL molecular subtypes and determining biological signatures and microenvironment but routine use of this technology has not been adopted widely for diagnostic practice.^45,61^

## WHEN IS A DIAGNOSIS OF FOLLICULAR HELPER T-CELL LYMPHOMA APPROPRIATE?

It is helpful when knowledge of the clinical presentation including coexisting disorders and chronic infections and laboratory findings are available to the pathologist. A characteristic clinical syndrome of autoimmune manifestations, disseminated lymphadenopathy, skin rashes, effusions, and B symptoms may help to guide the diagnosis.^83^ Exclusion of reactive causes of TFH proliferation including drug reactions, vaccinations, viral infections, and other immune conditions is necessary, and exclusion of nodal involvement by cutaneous T-cell lymphoma is required by the ICC and WHO5 classification schemes.^1,4^ Serology testing for human lymphotropic virus 1 (HTLV1) should be performed in principle, because up to 30% of ATLLs express TFH markers to some extent.^84–86^

**FIGURE 14:**
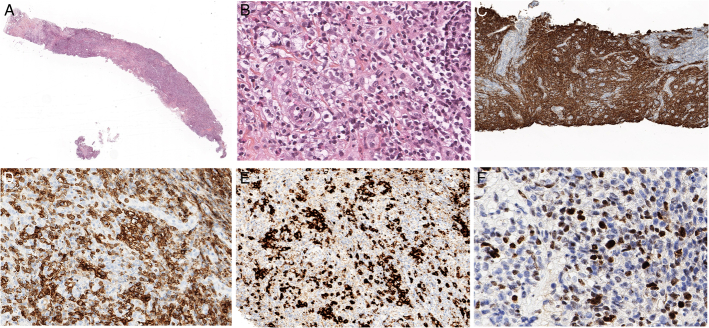
Core needle biopsy with follicular helper T-cell lymphoma, angioimmunoblastic type. This core needle biopsy of relatively good size (A) shows prominent vessels and a polymorphous infiltrate including lymphoid cells with clear cytoplasm (B). C, CD23 highlights a diffuse and dense meshwork of follicular dendritic cells which is key in establishing the diagnosis. The atypical cells are positive for CD5 (D), with expression of CD10 (E) and BCL6 (F).

TFHL is comprised of 3 morphologic subtypes: AI-type, follicular-type, and not otherwise specified. Recognition of the histologic pattern of these types will assist in diagnosis. AITL-type is composed of a polymorphous infiltrate including variable proportions of neoplastic cells, with intermixed small lymphocytes, histiocytes, immunoblasts, eosinophils, and plasma cells. The abnormal lymphoid cells are usually small to medium in size with nuclear atypia and clear cytoplasm. There is usually a stromal component with arborizing high endothelial venules and typically disrupted and proliferated follicular dendritic cell (FDC) meshworks (Figs. 13C, 14). In some cases, histiocytes, large B-cell immunoblasts or HRS-like cells might be abundant.^29,68,87^ TFHL follicular type is least common and is comprised of pale aggregates of medium-sized abnormal T cells within expanded IgD+ mantle zones (progressive transformation of germinal center-like pattern) or resembles a follicular lymphoma-like pattern with nodular aggregates of neoplastic cells with an abnormal TFH T-cell phenotype within a meshwork of follicular dendritic cells. These cases lack the extrafollicular proliferation of FDC and increased vascular density of AITL-type.^88,89^ TFH lymphoma, NOS, fits the definition of TFHL by immunophenotype and may have some characteristics of AITL-type, but may present as what was formerly called the “T-zone variant” of PTCL, NOS with a TFH immunophenotype, or with perifollicular involvement, or with an entirely diffuse growth pattern without the FDC meshworks characteristic of AITL-type.^68,90^

## WHAT CHARACTERISTICS ARE MOST USEFUL TO DIFFERENTIATE ANAPLASTIC LARGE CELL LYMPHOMA FROM PERIPHERAL T-CELL LYMPHOMA, NOT OTHERWISE SPECIFIED?

**FIGURE 15:**
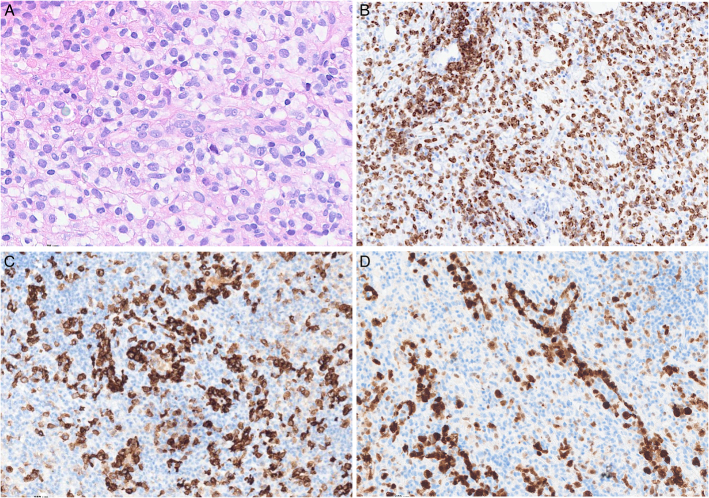
Small-cell pattern of ALK+ anaplastic large cell lymphoma. A, The neoplastic infiltrate consists of small to medium-sized cells with clear cytoplasm and scattered larger cells. The atypical cells are positive for CD3 (B), CD30 stains strongly the perivascular cells and is weak to negative in the other cells (C); similarly, ALK is mostly expressed in the cells surrounding the vessels (D).

It is necessary to carefully consider how to differentiate ALCL and PTCL, NOS. Patients with CD30+ PTCL, NOS appear to have an inferior prognosis than patients with ALCL, ALK-.^91^ Some cases of PTCL NOS can express CD30 strongly in a substantial proportion of cells. In one study of 141 PTCL NOS, more than 20% of cases showed CD30 positivity in >50% of the tumor cells.^92^ The small cell variant of ALCL, ALK+ (5% to 10% of cases of ALK+ ALCL) follows a different CD30 expression pattern than other variants of ALCL and can be confused with PTCL, NOS if immunohistochemistry for ALK is not performed. A minority of the neoplastic cells are large with hallmark morphology, and these tend to cluster in a perivascular location (Fig. 15). Some of the lymphoma cells are quite small and weak to negative for CD30, which could lead to a mistaken diagnosis of PTCL NOS. For this reason, ALK immunohistochemistry must be applied in any case of T-cell lymphoma with any degree of CD30 expression.^38^

A subgroup of CD30+ PTCLs with anaplastic morphology and Hodgkin-like features including a polymorphous inflammatory background and eosinophilia originally described in 2003 may cause confusion with classic Hodgkin lymphoma or other CD30 positive T-cell lymphomas.^93^ Similar cases were later found to harbor recurrent *JAK2* rearrangements.^94^ In a different study, a cohort of CD30+CD15+ PTCL, NOS were compared with ALK- ALCL by RNA sequencing and showed no clear segregation between the 2 groups, and a subset of PTCL, NOS cases demonstrated *DUSP22* rearrangements by FISH, suggesting that these cases may fit better within the spectrum of ALK- ALCL than PTCL, NOS.^83,95^

While *TP63* rearrangements have been described with prognostic association in a subset of ALCL, ALK-,^40^ *TP63* rearrangements have been reported in both ALCL, ALK- and PTCL, NOS, so this finding does not necessarily favor ALK- ALCL if other features are equivalent.^94^ Some cases of PTCL, NOS with a cytotoxic phenotype have *IRF4* rearrangements [resulting from t(6;14)(p25;q11.2)] that may be detected using a FISH probe for *DUSP22/IRF4* locus. These are CD30-negative and should not be confused with *DUSP22*-R ALK- ALCL.^97^

## CAN NODAL PERIPHERAL T-CELL LYMPHOMA BE ACCURATELY DIAGNOSED ON NEEDLE BIOPSIES?

Core needle biopsy lymph node samples (CNB) are a viable option and are increasingly performed for reasons of cost, waiting time for the procedure, and less morbidity. Nonetheless, obtaining material for lymphoma diagnosis through a surgical excisional biopsy is considered ideal because collection of more tissue allows the assessment of architecture, performance of immunohistochemistry and molecular tests, and residual archival tissue that can be used later for research or clinical trial enrollment. A study performed by the French Lymphopath Network involving a large inventory of lymph node CNB and surgical excision samples from patients with suspected lymphoma, from 2010 to 2018, saw an increase in CNB from 25% to 40% during the study period. Although CNB provided a definitive diagnosis in most of the patients with suspected lymphoma, it showed an increased risk of nondefinitive diagnosis with CNB samples and suggested the need for expert review in cases of lymphoma suspicion in CNB specimens. The diagnostic performance of expert pathology review was higher in excisional biopsies compared with CNB.^98,99^ Another study performed by colleagues in Brazil identified a high rate of definitive diagnosis with CNB but an increased percentage of nondiagnostic biopsies in T-cell lymphomas (30%), followed by classic Hodgkin lymphoma (10.6%).^100^ The polymorphic background and sparse large HRS cells in HL, and the microenvironmental features in TFHL (which can occasionally contain HRS-like cells) can be difficult to evaluate on CNB without careful assessment of the lymph node architecture. The growth pattern of TFHL AI-type often requires an expanded meshwork of follicular dendritic cells (Fig. 14) and evaluation of the distribution of TFH cells within the context of paracortex and follicles.^98^

CNB may not always provide a faster diagnosis due to the risk of one or several procedural attempts before achieving adequate material for diagnosis. Adequate CNB sampling including a wide gauge needle and multiple cores may be sufficient for diagnostic testing in the majority of cases and might even allow for molecular tests including high-throughput sequencing. However, some patients with a final diagnosis of TFHL made on excision may have one or several needle core biopsies preceding the contributive excisional biopsy. TFHL on final excisional biopsy may be preceded by an erroneous diagnosis (HL, EBV+ B-cell lymphoproliferative disorder) or a nonconclusive diagnosis as seen in a small cohort of workshop cases.^18^

In CNB samples of suspected lymphoma that demonstrate diffuse growth with a monomorphic large cell morphology and abnormal T-cell immunophenotype, a diagnosis of a specific T-cell lymphoma entity may be possible. Other CNB samples with a heterogeneous background, limited visual assessment of lymph node architecture, poor conceptualization of follicular dendritic cell meshwork patterns, or samples containing large HRS-like cells are problematic and a diagnosis of atypical lymphoproliferation (more or less suspicious for malignancy) may be advisable with the recommendation to perform an excisional biopsy.

## CONCLUSION

When encountering a patient with suspected nodal T-cell lymphoma, the stepwise approach derived from recent classification schemes provide a useful framework for diagnosis. Morphologic assessment complemented by immunophenotypic and molecular studies increasingly incorporated in the routine practice remain the mainstay of pathologic routine diagnosis. Considering pitfalls and benign mimics, surgical biopsies are the preferred sampling method. In many instances, final diagnoses require integrating the pathologic findings with clinical features, particularly the disease distribution, and the presence of extranodal or cutaneous involvement.

## References

[1] Campo E Jaffe ES Cook JR . The International Consensus Classification of Mature Lymphoid Neoplasms: a report from the Clinical Advisory Committee. Blood. 2022;140:1229–1253. 35653592 10.1182/blood.2022015851 PMC9479027

[2] Feldman AL Laurent C Narbaitz M . Classification and diagnostic evaluation of nodal T- and NK-cell lymphomas. Virchows Arch. 2023;482:265–279. 36210383 10.1007/s00428-022-03412-6

[3] Alaggio R Amador C Anagnostopoulos I . The 5th edition of the World Health Organization Classification of Haematolymphoid Tumours: Lymphoid Neoplasms. Leukemia. 2022;36:1720–1748. 35732829 10.1038/s41375-022-01620-2 PMC9214472

[4] Board. WCoHTE . *Haematolymphoid Tumors* Vol 11 5th ed. International Agency for Research on Cancer; 2024.

[5] Amador C Chan WC . Nodal peripheral T-cell lymphomas in the new classification systems. Cancer Biol Med. 2024;20:922–926. 38318921 10.20892/j.issn.2095-3941.2023.0490 PMC10845937

[6] Tzankov A Dirnhofer S . A pattern-based approach to reactive lymphadenopathies. Semin Diagn Pathol. 2018;35:4–19. 28619469 10.1053/j.semdp.2017.05.002

[7] de Leval L . Approach to nodal-based T-cell lymphomas. Pathology. 2020;52:78–99. 31785821 10.1016/j.pathol.2019.09.012

[8] Weiss LM O’Malley D . Benign lymphadenopathies. Mod Pathol. 2013;26(suppl 1):S88–S96. 23281438 10.1038/modpathol.2012.176

[9] Louissaint A Jr Ferry JA Soupir CP . Infectious mononucleosis mimicking lymphoma: distinguishing morphological and immunophenotypic features. Mod Pathol. 2012;25:1149–1159. 22627742 10.1038/modpathol.2012.70

[10] Malik UR Oleksowicz L Dutcher JP . Atypical clonal T-cell proliferation in infectious mononucleosis. Med Oncol. 1996;13:207–213. 9152971 10.1007/BF02990933

[11] Abbondazo SL Irey NS Frizzera G . Dilantin-associated lymphadenopathy. Spectrum of histopathologic patterns. Am J Surg Pathol. 1995;19:675–686. 7755154 10.1097/00000478-199506000-00008

[12] Ben M’rad M Leclerc-Mercier S Blanche P . Drug-induced hypersensitivity syndrome: clinical and biologic disease patterns in 24 patients. Medicine (Baltimore). 2009;88:131–140. 19440116 10.1097/MD.0b013e3181a4d1a1

[13] Bledsoe JR Ferry JA Neyaz A . IgG4-related lymphadenopathy: a comparative study of 41 cases reveals distinctive histopathologic features. Am J Surg Pathol. 2021;45:178–192. 32889888 10.1097/PAS.0000000000001579

[14] Attygalle AD Kyriakou C Dupuis J . Histologic evolution of angioimmunoblastic T-cell lymphoma in consecutive biopsies: clinical correlation and insights into natural history and disease progression. Am J Surg Pathol. 2007;31:1077–1088. 17592275 10.1097/PAS.0b013e31802d68e9

[15] Lim MS Straus SE Dale JK . Pathological findings in human autoimmune lymphoproliferative syndrome. Am J Pathol. 1998;153:1541–1550. 9811346 10.1016/S0002-9440(10)65742-2 PMC1853411

[16] Onciu M Medeiros LJ . Kikuchi-Fujimoto lymphadenitis. Adv Anat Pathol. 2003;10:204–211. 12826826 10.1097/00125480-200307000-00003

[17] Ree H Kadin M Kikuchi M . Angioimmunoblastic lymphoma (AILD-type T-cell lymphoma) with hyperplastic germinal centers. Am J Surg Pathol. 1998;22:643–655. 9630171 10.1097/00000478-199806000-00001

[18] Ondrejka SL Amador C Climent F . Follicular helper T-cell lymphomas: disease spectrum, relationship with clonal hematopoiesis, and mimics. A report of the 2022 EA4HP/SH lymphoma workshop. Virchows Arch. 2023;483:349–365. 37500795 10.1007/s00428-023-03607-5 PMC10541838

[19] Vallangeon BKA Tissue disaggregation methods for flow cytometric immunophenotyping. ICCS Qual Standards Committee. 2019.

[20] Aggarwal N Fischer J Swerdlow SH . Splenic lymphoid subsets with less well-recognized phenotypes mimic aberrant antigen expression. Am J Clin Pathol. 2013;140:787–794. 24225744 10.1309/AJCPPIBH3I1VRWXQ

[21] Jevremovic D Olteanu H . Flow cytometry applications in the diagnosis of T/NK-cell lymphoproliferative disorders. Cytometry B Clin Cytom. 2019;96:99–115. 30729667 10.1002/cyto.b.21768

[22] Jamal S Picker LJ Aquino DB . Immunophenotypic analysis of peripheral T-cell neoplasms. A multiparameter flow cytometric approach. Am J Clin Pathol. 2001;116:512–526. 11601136 10.1309/QF6N-VAQW-N74H-4JE2

[23] Demurtas A Stacchini A Aliberti S . Tissue flow cytometry immunophenotyping in the diagnosis and classification of non-Hodgkin’s lymphomas: a retrospective evaluation of 1,792 cases. Cytometry B Clin Cytom. 2013;84B:82–95. 10.1002/cyto.b.21065 23325563

[24] Loghavi S Wang SA Medeiros LJ . Immunophenotypic and diagnostic characterization of angioimmunoblastic T-cell lymphoma by advanced flow cytometric technology. Leuk Lymphoma. 2016;57:2804–2812. 27105079 10.3109/10428194.2016.1170827 PMC5142610

[25] Baseggio L Traverse-Glehen A Berger F . CD10 and ICOS expression by multiparametric flow cytometry in angioimmunoblastic T-cell lymphoma. Mod Pathol. 2011;24:993–1003. 21499231 10.1038/modpathol.2011.53

[26] Chen W Kesler MV Karandikar NJ . Flow cytometric features of angioimmunoblastic T-cell lymphoma. Cytometry B Clin Cytom. 2006;70:142–148. 16572417 10.1002/cyto.b.20107

[27] Alikhan M Song JY Sohani AR . Peripheral T-cell lymphomas of follicular helper T-cell type frequently display an aberrant CD3(-/dim)CD4(+) population by flow cytometry: an important clue to the diagnosis of a Hodgkin lymphoma mimic. Mod Pathol. 2016;29:1173–1182. 27312067 10.1038/modpathol.2016.113 PMC6331059

[28] Hoffmann JC Chisholm KM Cherry A . An analysis of MYC and EBV in diffuse large B-cell lymphomas associated with angioimmunoblastic T-cell lymphoma and peripheral T-cell lymphoma not otherwise specified. Hum Pathol. 2016;48:9–17. 26772393 10.1016/j.humpath.2015.09.033

[29] Xie Y Jaffe ES . How I diagnose angioimmunoblastic T-cell lymphoma. Am J Clin Pathol. 2021;156:1–14. 34117736 10.1093/ajcp/aqab090 PMC8209595

[30] Berg H Otteson GE Corley H . Flow cytometric evaluation of TRBC1 expression in tissue specimens and body fluids is a novel and specific method for assessment of T-cell clonality and diagnosis of T-cell neoplasms. Cytometry B Clin Cytom. 2021;100:361–369. 32333725 10.1002/cyto.b.21881

[31] Horna P Weybright MJ Ferrari M . Dual T-cell constant β chain (TRBC)1 and TRBC2 staining for the identification of T-cell neoplasms by flow cytometry. Blood Cancer J. 2024;14:34. 38424120 10.1038/s41408-024-01002-0 PMC10904869

[32] Shi M Jevremovic D Otteson GE . Single antibody detection of T-cell receptor αβ clonality by flow cytometry rapidly identifies mature T-cell neoplasms and monotypic small CD8-positive subsets of uncertain significance. Cytometry B Clin Cytom. 2020;98:99–107. 30972977 10.1002/cyto.b.21782

[33] Devitt KA Kern W Li W . TRBC1 in flow cytometry: assay development, validation, and reporting considerations. Cytometry B Clin Cytom. 2024;106:192–202. 38700195 10.1002/cyto.b.22175

[34] Wadsworth P Zhang J Miller T . Prevalence and clinicopathological features of incidentally detected TRBC1-dim populations in peripheral blood flow cytometry. Leuk Lymphoma. 2024;65:1374–1377; 1–4. 38747176 10.1080/10428194.2024.2354527

[35] Khanlari M Ramos JC Sanchez SP . Adult T-cell leukemia/lymphoma can be indistinguishable from other more common T-cell lymphomas. The University of Miami experience with a large cohort of cases. Mod Pathol. 2018;31:1046–1063. 29449683 10.1038/s41379-018-0037-3 PMC6931282

[36] Hsi ED Said J Macon WR . Diagnostic accuracy of a defined immunophenotypic and molecular genetic approach for peripheral T/NK-cell lymphomas. A North American PTCL study group project. Am J Surg Pathol. 2014;38:768–775. 24618604 10.1097/PAS.0000000000000188 PMC4085049

[37] Gru AA Lim MS Dogan A . Best practices in CD30 immunohistochemistry testing, interpretation, and reporting: an expert panel consensus. Arch Pathol Lab Med. 2023;147:79–86. 35472771 10.5858/arpa.2021-0270-OA

[38] Vega F Medeiros LJ . A suggested immunohistochemical algorithm for the classification of T-cell lymphomas involving lymph nodes. Hum Pathol. 2020;102:104–116. 32479842 10.1016/j.humpath.2020.05.006

[39] Chiattone C Civallero M Fischer T . Characteristics and clinical outcomes of patients with ALK-positive anaplastic large cell lymphoma: report from the prospective international T-cell lymphoma project. Hematol Oncol. 2022;40:953–961. 36083035 10.1002/hon.3074

[40] Parrilla Castellar ER Jaffe ES Said JW . ALK-negative anaplastic large cell lymphoma is a genetically heterogeneous disease with widely disparate clinical outcomes. Blood. 2014;124:1473–1480. 24894770 10.1182/blood-2014-04-571091 PMC4148769

[41] King RL Dao LN McPhail ED . Morphologic features of ALK-negative anaplastic large cell lymphomas with DUSP22 rearrangements. Am J Surg Pathol. 2016;40:36–43. 26379151 10.1097/PAS.0000000000000500 PMC4834837

[42] Ravindran A Feldman AL Ketterling RP . Striking association of lymphoid enhancing factor (LEF1) overexpression and DUSP22 rearrangements in anaplastic large cell lymphoma. Am J Surg Pathol. 2021;45:550–557. 33165091 10.1097/PAS.0000000000001614

[43] Amador C Feldman AL . How I diagnose anaplastic large cell lymphoma. Am J Clin Pathol. 2021;155:479–497. 33686426 10.1093/ajcp/aqab012

[44] Pletneva MA Smith LB . Anaplastic large cell lymphoma: features presenting diagnostic challenges. Arch Pathol Lab Med. 2014;138:1290–1294. 25268191 10.5858/arpa.2014-0295-CC

[45] de Leval L Rickman DS Thielen C . The gene expression profile of nodal peripheral T-cell lymphoma demonstrates a molecular link between angioimmunoblastic T-cell lymphoma (AITL) and follicular helper T (TFH) cells. Blood. 2007;109:4952–4963. 17284527 10.1182/blood-2006-10-055145

[46] de Leval L Savilo E Longtine J . Peripheral T-cell lymphoma with follicular involvement and a CD4+/bcl-6+ phenotype. Am J Surg Pathol. 2001;25:395–400. 11224611 10.1097/00000478-200103000-00015

[47] Lemonnier F Couronné L Parrens M . Recurrent TET2 mutations in peripheral T-cell lymphomas correlate with TFH-like features and adverse clinical parameters. Blood. 2012;120:1466–1469. 22760778 10.1182/blood-2012-02-408542

[48] Palomero T Couronné L Khiabanian H . Recurrent mutations in epigenetic regulators, RHOA and FYN kinase in peripheral T cell lymphomas. Nat Genet. 2014;46:166–170. 24413734 10.1038/ng.2873 PMC3963408

[49] Dobay MP Lemonnier F Missiaglia E . Integrative clinicopathological and molecular analyses of angioimmunoblastic T-cell lymphoma and other nodal lymphomas of follicular helper T-cell origin. Haematologica. 2017;102:e148–e151. 28082343 10.3324/haematol.2016.158428 PMC5395128

[50] Basha BM Bryant SC Rech KL . Application of a 5 marker panel to the routine diagnosis of peripheral T-cell lymphoma with T-follicular helper phenotype. Am J Surg Pathol. 2019;43:1282–1290. 31283630 10.1097/PAS.0000000000001315

[51] Rodríguez-Pinilla SM Atienza L Murillo C . Peripheral T-cell lymphoma with follicular T-cell markers. Am J Surg Pathol. 2008;32:1787–1799. 18779728 10.1097/PAS.0b013e31817f123e

[52] Krishnan C Warnke RA Arber DA . PD-1 expression in T-cell lymphomas and reactive lymphoid entities: potential overlap in staining patterns between lymphoma and viral lymphadenitis. Am J Surg Pathol. 2010;34:178–189. 20087161 10.1097/PAS.0b013e3181cc7e79 PMC2819320

[53] Gaulard P de Leval L . Follicular helper T cells: implications in neoplastic hematopathology. Semin Diagn Pathol. 2011;28:202–213. 21850986 10.1053/j.semdp.2011.03.003 PMC4019516

[54] de Leval L Gisselbrecht C Gaulard P . Advances in the understanding and management of angioimmunoblastic T-cell lymphoma. Br J Haematol. 2010;148:673–689. 19961485 10.1111/j.1365-2141.2009.08003.x

[55] Abukhiran I Syrbu SI Holman CJ . Markers of follicular helper T cells are occasionally expressed in T-cell or histiocyte-rich large B-cell lymphoma, classic Hodgkin lymphoma, and atypical paracortical hyperplasia: a diagnostic pitfall for T-cell lymphomas of T follicular helper origin. Am J Clin Pathol. 2021;156:409–426. 33624021 10.1093/ajcp/aqaa249

[56] Egan C Laurent C Alejo JC . Expansion of PD1-positive T cells in nodal marginal zone lymphoma: a potential diagnostic pitfall. Am J Surg Pathol. 2020;44:657–664. 31764221 10.1097/PAS.0000000000001414 PMC8189156

[57] Kanavaros P Boulland ML Petit B . Expression of cytotoxic proteins in peripheral T-cell and natural killer-cell (NK) lymphomas: association with extranodal site, NK or Tgammadelta phenotype, anaplastic morphology and CD30 expression. Leuk Lymphoma. 2000;38:317–326. 10830738 10.3109/10428190009087022

[58] Asano N Suzuki R Kagami Y . Clinicopathologic and prognostic significance of cytotoxic molecule expression in nodal peripheral T-cell lymphoma, unspecified. Am J Surg Pathol. 2005;29:1284–1293. 16160469 10.1097/01.pas.0000173238.17331.6b

[59] Nicolae A Bouilly J Lara D . Nodal cytotoxic peripheral T-cell lymphoma occurs frequently in the clinical setting of immunodysregulation and is associated with recurrent epigenetic alterations. Mod Pathol. 2022;35:1126–1136. 35301414 10.1038/s41379-022-01022-w

[60] Amador C Greiner TC Heavican TB . Reproducing the molecular subclassification of peripheral T-cell lymphoma-NOS by immunohistochemistry. Blood. 2019;134:2159–2170. 31562134 10.1182/blood.2019000779 PMC6908831

[61] Iqbal J Wright G Wang C . Gene expression signatures delineate biological and prognostic subgroups in peripheral T-cell lymphoma. Blood. 2014;123:2915–2923. 24632715 10.1182/blood-2013-11-536359 PMC4014836

[62] Heavican TB Bouska A Yu J . Genetic drivers of oncogenic pathways in molecular subgroups of peripheral T-cell lymphoma. Blood. 2019;133:1664–1676. 30782609 10.1182/blood-2018-09-872549 PMC6460420

[63] Amador C Weisenburger DD Gomez A . Refining diagnostic subtypes of peripheral T-cell lymphoma using a multiparameter approach. Mod Pathol. 2024;38:100646. 39491745 10.1016/j.modpat.2024.100646 PMC11845303

[64] Ohgami RS Arber DA Zehnder JL . Indolent T-lymphoblastic proliferation (iT-LBP): a review of clinical and pathologic features and distinction from malignant T-lymphoblastic lymphoma. Adv Anat Pathol. 2013;20:137–140. 23574769 10.1097/PAP.0b013e31828d17ec

[65] Climent F Nicolae A de Leval L . Cytotoxic peripheral T-cell lymphomas and EBV-positive T/NK-cell lymphoproliferative diseases: emerging concepts, recent advances, and the putative role of clonal hematopoiesis. A report of the 2022 EA4HP/SH lymphoma workshop. Virchows Arch. 2023;483:333–348. 37646869 10.1007/s00428-023-03616-4 PMC10542298

[66] Kato S Asano N Miyata-Takata T . T-cell receptor (TCR) phenotype of nodal Epstein-Barr virus (EBV)-positive cytotoxic T-cell lymphoma (CTL): a clinicopathologic study of 39 cases. Am J Surg Pathol. 2015;39:462–471. 25634749 10.1097/PAS.0000000000000323

[67] Jeon YK Kim JH Sung JY . Epstein-Barr virus-positive nodal T/NK-cell lymphoma: an analysis of 15 cases with distinct clinicopathological features. Hum Pathol. 2015;46:981–990. 25907865 10.1016/j.humpath.2015.03.002

[68] Bisig B Savage KJ De Leval L . Pathobiology of nodal peripheral T-cell lymphomas: current understanding and future directions. Haematologica. 2023;108:3227–3243. 38037800 10.3324/haematol.2023.282716 PMC10690915

[69] Li S Feng X Li T . Extranodal NK/T-cell lymphoma, nasal type: a report of 73 cases at MD Anderson Cancer Center. Am J Surg Pathol. 2013;37:14–23. 23232851 10.1097/PAS.0b013e31826731b5

[70] Hartmann S Goncharova O Portyanko A . CD30 expression in neoplastic T cells of follicular T cell lymphoma is a helpful diagnostic tool in the differential diagnosis of Hodgkin lymphoma. Mod Pathol. 2019;32:37–47. 30140037 10.1038/s41379-018-0108-5

[71] Nicolae A Pittaluga S Venkataraman G . Peripheral T-cell lymphomas of follicular T-helper cell derivation with Hodgkin/Reed-Sternberg cells of B-cell lineage: both EBV-positive and EBV-negative variants exist. Am J Surg Pathol. 2013;37:816–826. 23598959 10.1097/PAS.0b013e3182785610 PMC3654051

[72] Zettl A Lee S-S Rüdiger T . Epstein-Barr virus-associated B-cell lymphoproliferative disorders in angioimmunoblastic T-cell lymphoma and peripheral T-cell lymphoma, unspecified. Am J Clin Pathol. 2002;117:368–379. 11888076 10.1309/6UTX-GVC0-12ND-JJEU

[73] Balagué O Martínez A Colomo L . Epstein-Barr virus negative clonal plasma cell proliferations and lymphomas in peripheral T-cell lymphomas: a phenomenon with distinctive clinicopathologic features. Am J Surg Pathol. 2007;31:1310–1322. 17721185 10.1097/PAS.0b013e3180339f18

[74] Van Krieken J Langerak A Macintyre E . Improved reliability of lymphoma diagnostics via PCR-based clonality testing: report of the BIOMED-2 Concerted Action BHM4-CT98-3936. Leukemia. 2007;21:201–206. 17170732 10.1038/sj.leu.2404467

[75] Langerak A Molina T Lavender F . PCR-based clonality testing in tissue samples with reactive lymphoproliferations: usefulness and pitfalls. A study from the BIOMED-2 Concerted Action BMH4-CT98-3936. Leukemia. 2007;21:222–229. 17170729 10.1038/sj.leu.2404482

[76] Qayyum S Bullock GC Swerdlow SH . Diagnostic utility of isolated tube C positivity in T-cell receptor β testing using BIOMED-2 primers. Am J Clin Pathol. 2019;151:386–394. 30534953 10.1093/ajcp/aqy157

[77] Donelli R Gazzola A Mannu C . Conventional PCR-based versus next-generation sequencing-based approach for T-cell receptor γ gene clonality assessment in mature T-cell lymphomas: a phase 3 diagnostic accuracy study. J Biologic Methods. 2024;11:e99010013. 10.14440/jbm.2024.0002 PMC11423944 39323485

[78] Stewart JP Gazdova J Darzentas N . Validation of the EuroClonality-NGS DNA capture panel as an integrated genomic tool for lymphoproliferative disorders. Blood Adv. 2021;5:3188–3198. 34424321 10.1182/bloodadvances.2020004056 PMC8405189

[79] de Leval L Alizadeh AA Bergsagel PL . Genomic profiling for clinical decision making in lymphoid neoplasms. Blood. 2022;140:2193–2227. 36001803 10.1182/blood.2022015854 PMC9837456

[80] Couronné L Bastard C Bernard OA . TET2 and DNMT3A mutations in human T-cell lymphoma. N Engl J Med. 2012;366:95–96. 22216861 10.1056/NEJMc1111708

[81] Lewis NE Petrova-Drus K Huet S . Clonal hematopoiesis in angioimmunoblastic T-cell lymphoma with divergent evolution to myeloid neoplasms. Blood Adv. 2020;4:2261–2271. 32442302 10.1182/bloodadvances.2020001636 PMC7252546

[82] Yao WQ Wu F Zhang W . Angioimmunoblastic T-cell lymphoma contains multiple clonal T-cell populations derived from a common TET2 mutant progenitor cell. J Pathol. 2020;250:346–357. 31859368 10.1002/path.5376 PMC7064999

[83] de Leval LL Gaulard P Dogan A . A practical approach to the modern diagnosis and classification of T-and NK-cell lymphomas. Blood. 2024;144:1855–1872. 38728419 10.1182/blood.2023021786 PMC11830980

[84] Horwitz SM Ansell S Ai WZ . T-cell lymphomas, version 2.2022, NCCN clinical practice guidelines in oncology. J Natl Compr Cancer Netw. 2022;20:285–308. 10.6004/jnccn.2022.0015 35276674

[85] Muto R Miyoshi H Nakashima K . Clinicopathological features of adult T‐cell leukemia/lymphoma with T‐follicular helper phenotype. Cancer Med. 2024;13:e7050. 38506241 10.1002/cam4.7050 PMC10952016

[86] Tamaki T Karube K Sakihama S . A Comprehensive study of the immunophenotype and its clinicopathologic significance in adult T-Cell leukemia/lymphoma. Mod Pathol. 2023;36:100169. 36997002 10.1016/j.modpat.2023.100169

[87] Hartmann S Agostinelli C Klapper W . Revising the historical collection of epithelioid cell-rich lymphomas of the Kiel Lymph Node Registry: what is Lennert’s lymphoma nowadays? Histopathology. 2011;59:1173–1182. 22175897 10.1111/j.1365-2559.2011.04069.x

[88] Huang Y Moreau A Dupuis J . Peripheral T-cell lymphomas with a follicular growth pattern are derived from follicular helper T cells (TFH) and may show overlapping features with angioimmunoblastic T-cell lymphomas. Am J Surg Pathol. 2009;33:682–690. 19295409 10.1097/PAS.0b013e3181971591 PMC4838638

[89] Miyoshi H Sato K Niino D . Clinicopathologic analysis of peripheral T-cell lymphoma, follicular variant, and comparison with angioimmunoblastic T-cell lymphoma: Bcl-6 expression might affect progression between these disorders. Am J Clin Pathol. 2012;137:879–889. 22586046 10.1309/AJCPBPNV86VZENGV

[90] Agostinelli C Hartmann S Klapper W . Peripheral T cell lymphomas with follicular T helper phenotype: a new basket or a distinct entity? Revising Karl Lennert’s personal archive. Histopathology. 2011;59:679–691. 22014049 10.1111/j.1365-2559.2011.03981.x

[91] Savage KJ Harris NL Vose JM . ALK− anaplastic large-cell lymphoma is clinically and immunophenotypically different from both ALK+ ALCL and peripheral T-cell lymphoma, not otherwise specified: report from the International Peripheral T-Cell Lymphoma Project. Blood. 2008;111:5496–5504. 18385450 10.1182/blood-2008-01-134270

[92] Bossard C Dobay MP Parrens M . Immunohistochemistry as a valuable tool to assess CD30 expression in peripheral T-cell lymphomas: high correlation with mRNA levels. Blood. 2014;124:2983–2986. 25224410 10.1182/blood-2014-07-584953

[93] Barry TS Jaffe ES Sorbara L . Peripheral T-cell lymphomas expressing CD30 and CD15. Am J Surg Pathol. 2003;27:1513–1522. 14657710 10.1097/00000478-200312000-00003

[94] Fitzpatrick MJ Massoth LR Marcus C . JAK2 rearrangements are a recurrent alteration in CD30+ systemic T-cell lymphomas with anaplastic morphology. Am J Surg Pathol. 2021;45:895–904. 34105517 10.1097/PAS.0000000000001708

[95] Ganapathi KA Nicolae A Egan C . Peripheral T-cell lymphomas expressing CD30 and CD15 expand the spectrum of anaplastic large cell lymphoma, ALK-negative. Br J Haematol. 2024;204:1862–1871. 38613165 10.1111/bjh.19442

[96] Vasmatzis G Johnson SH Knudson RA . Genome-wide analysis reveals recurrent structural abnormalities of TP63 and other p53-related genes in peripheral T-cell lymphomas. Blood. 2012;120:2280–2289. 22855598 10.1182/blood-2012-03-419937 PMC5070713

[97] Feldman AL Law M Remstein ED . Recurrent translocations involving the IRF4 oncogene locus in peripheral T-cell lymphomas. Leukemia. 2009;23:574–580. 18987657 10.1038/leu.2008.320 PMC2656414

[98] Syrykh C Chaouat C Poullot E . Lymph node excisions provide more precise lymphoma diagnoses than core biopsies: a French Lymphopath network survey. Blood. 2022;140:2573–2583. 35797472 10.1182/blood.2022015520

[99] Jaffe ES Cook JR . Core biopsy for lymphoma diagnosis? A needling prospect. Blood. 2022;140:2525–2527. 36520478 10.1182/blood.2022017461

[100] Gonçalves MC de Oliveira C Sandes AF . Core needle biopsy in lymphoma diagnosis: the diagnostic performance and the role of the multidisciplinary approach in the optimization of results. Am J Surg Pathol. 2023;47:111–123. 36395467 10.1097/PAS.0000000000001991

